# Immune-related adverse events in the treatment of non-Hodgkin lymphoma with immune checkpoint inhibitors

**DOI:** 10.1038/s41598-022-05861-0

**Published:** 2022-02-02

**Authors:** Lisa Argnani, Beatrice Casadei, Carla Pelusi, Valentina Lo Preiato, Uberto Pagotto, Francesco Bertoni, Pier Luigi Zinzani

**Affiliations:** 1 Istituto di Ematologia “Seràgnoli”, IRCCS Azienda Ospedaliero-Universitaria di Bologna, Via Massarenti 9, 40138 Bologna, Italy; 2 Dipartimento di Medicina Specialistica, Diagnostica e Sperimentale, Università di Bologna, Bologna, Italy; 3 Unit of Endocrinology and Prevention and Care of Diabetes, IRCCS Azienda Ospedaliero-Universitaria di Bologna, University of Bologna, Bologna, Italy; 4 Faculty of Biomedical Sciences, Institute of Oncology Research, USI, Bellinzona, Switzerland; 5 Oncology Institute of Southern Switzerland, Bellinzona, Switzerland

**Keywords:** Non-hodgkin lymphoma, Medical research, Immunotherapy

## Abstract

Immune checkpoint inhibitors (ICIs) show efficacy in the treatment of non-Hodgkin lymphomas (NHL). However, these agents are associated with a unique group of side effects called immune-related adverse events (irAEs). We conducted an observational retrospective/prospective study on patients with relapsed/refractory NHL treated with ICI to determine the incidence of irAEs assessing the type, severity, and timing of onset, outcome and relationship with study drugs of these events. Thirty-two patients underwent ICI as single agent (N = 20) or in combination (N = 12). Ten patients (31.3%) developed at least one irAE for a total of 17 irAEs. Median time to presentation of irAEs was 69 days (range 0–407) with a median resolution time of 16 days (range 0–98). Progression free survival at 24 months for patients who developed an irAE was 40% and 31.8% for who did not. Overall survival for the two groups did not differ (at 24 months 40.0% and 62.5% for patients without and with irAE, respectively), but the median for who developed an irAE was not reached. The incidence of irAEs was associated with better long-term survival in NHL treated with ICIs but patients’ disease conditions need to be carefully evaluated to decide the optimal management.

## Introduction

Immune checkpoint inhibitors (ICIs), a new class of cancer therapeutic agents, seem to play an important role in the management of non-Hodgkin lymphomas (NHL) even though data are limited to clinical trials results^1–4^. ICIs have been approved for use in different malignancies including metastatic melanoma, advanced non-small cell lung cancer, metastatic renal cell carcinoma, refractory Hodgkin’s lymphoma, refractory primary mediastinal B-cell lymphoma (PMBCL, only by U.S. Food and Drug Administration), metastatic bladder cancer, and advanced head and neck cancer. For relapsed/refractory (R/R) NHL patients, who represent an unmet medical need, new encouraging results emerged for PMBCL, T-cell lymphomas and some B-cell lymphomas, even for rare extranodal ones^1,4–6^. However, specific toxicities related to ICIs are immune-related, differently from the side effects observed in previous oncologic treatment^7^. These immune-related adverse events (irAEs) are due to an excessive solicitation of the immune system—due to the mechanistic of each molecular target (i.e., cytotoxic T-Lymphocyte Antigen 4 [CTLA-4], and anti-programmed cell death 1 [PD-1] and anti-programmed cell death ligand 1 [PD-L1] network), may attack any organ and body district. In addition, irAEs can manifest at different timepoints both during treatment and even after the end of immunotherapy. IrAEs represent bystander effects from activated T-cells and it is plausible that patients responding to ICIs may present autoimmune toxicities (e.g. due to a more treatment-responsive immune system, or cross-reactivity between tumor and host tissue, or loss of central and peripheral tolerance towards self-antigens). Mild initial symptoms may suddenly become significantly worse and severe; therefore, it is extremely important to diagnose irAEs correctly, to determine their severity, and intervene correctly as soon as possible after their onset^7^. There is no prospective data on these toxicities, and guidelines or recommendations for lymphomas are currently based on symptomatic management from the ongoing clinical trials. Furthermore, guidelines are principally referred to solid neoplasia^7^. Hematologic irAEs induced by PD-1 or PD-L1 inhibitors are rare and potentially life-threatening. The most common clinical presentations are neutropenia, autoimmune hemolytic anemia, immune thrombocytopenia, and aplastic anemia^8^. Extra-hematologic irAEs are often low-grade and manageable, but they have the potential to be life-threatening and extremely severe if not promptly treated or managed incorrectly^9^. Among the irAEs, interstitial pneumonia, colitis, thyroiditis, hepatitis, skin rash, vitiligo, hypophysitis, autoimmune diabetes mellitus, renal dysfunction, myasthenia gravis, neuropathy, myositis, and uveitis are the most representative^7^.

Clinical issues emerged and include whether ICIs should be administered to patients with autoimmune disease and whether patients that develop irAEs should continue or not the immunotherapy. Furthermore, the onset of these irAEs events varies^7^. Key questions regarding the relationship between irAEs onset and ICIs efficacy remain. The most pertinent of these involve whether irAEs site, severity, timing of onset and management shape ICIs efficacy^10^.

In addition, the combination of immunotherapies in the near future means that hematologists will interface with a higher incidence and severity of irAEs.

Most of the data comes from solid neoplasia; there is little information on lymphomas, especially on NHL for which ICIs have not yet been approved worldwide.

Herein, we discuss our experience with ICIs in R/R NHL beginning to address these questions.

## Methods

An observational retrospective/prospective study was conducted on patients with R/R NHL treated with ICIs at our Institution. The study was approved by our local Ethical Committee (Comitato Etico Area Vasta Emilia Centrale di Bologna, IRCCS Azienda Ospedaliero-Universitaria di Bologna, approval id code 730/2019/Oss/AOUBo) and registered in the Italian Registry of Observational Studies. Patients provided signed informed consent, as applicable, in accordance with the Declaration of Helsinki. All methods were performed in accordance with the relevant and applicable guidelines and regulations.

Primary objective of the study was to determine the incidence of irAEs in patients affected by NHL undergoing ICIs treatment also assessing the type, severity, and timing of onset, management, outcome and relationship with study drugs of these events.

Secondary objectives were activity and disease control of ICIs along with their relationship with irAEs onset.

Patients remained in follow-up till the resolution of irAEs.

Objective response rate (ORR) was defined as the sum of complete response (CR) and partial response (PR) rates at the end of ICIs treatment and before any type of consolidation. Best response rate was defined as the sum of CR and PR rates reached at any time during treatment by each patient. Overall survival (OS) was defined as the time from initiation of therapy to death from any cause and was censored at the date of last available follow up. Progression free survival (PFS) was measured from initiation of therapy to progression, relapse, or death from any cause and was censored at the date of last available follow up. Disease free survival (DFS) was calculated for CR patients from the first documentation of response to the date of relapse or death due to lymphoma or acute toxicity of treatment^11^. Response was assessed using the International Working Group revised response criteria for malignant lymphoma^11,12^. Safety and tolerability were evaluated by recording incidence, severity, and type of any AE according to the National Cancer Institute Common Terminology Criteria for AEs v4.0. A minimum of 12 months of follow up was required for the analyses to evaluate late Aes.

Demographics and patients’ characteristics as well Aes were summarized by descriptive statistics. Continuous variables were reported as median (range) for non-normally distributed data and compared using the Student t-test or Mann Whitney U test. Categorical variables were reported as absolute and relative frequencies and compared using Fisher’s exact test or Chi-squared test, as applicable. Correlations were tested among irAEs occurrence, effectiveness of ICIs and patients’ survivals. Survival functions were estimated by using the Kaplan–Meier method. Statistical analyses were performed with Stata 11 (StataCorp LP, TX) and p values were set at 0.05.

### Ethical conduct of research statement

The study was approved by the local Ethical Committee (Comitato Etico Area Vasta Emilia Centrale di Bologna, IRCCS Azienda Ospedaliero-Universitaria di Bologna, approval id code 730/2019/Oss/AOUBo) and registered in the Italian Registry of Observational Studies. Patients provided signed informed consent, as applicable, in accordance with the Declaration of Helsinki.

## Results

Thirty-two NHL patients (12 males and 20 females) were enrolled. They were treated between September 2014 and February 2019. Patients had PMBCL (N = 26), mycosis fungoides/Sézary syndrome (N = 5) and follicular T-helper lymphoma (N = 1).

Median age at diagnosis was 31 years (range, 19–61). Patients had a median of 3 previous therapies (range 1–9), including autologous stem cell transplantation (ASCT, N = 6) and brentuximab vedotin (BV, N = 5). Twenty-nine patients were refractory to first-line treatment and 29 to the last one.

Eighteen patients underwent pembrolizumab, 12 had nivolumab in combination with BV, 1 patient underwent nivolumab and 1 received tislelizumab, with a median of 5 cycles (1–52).

No dose reduction for ICIs have been necessary (only 1 reduction and subsequent discontinuation of BV) and 21 patients had an early drug discontinuation: 18 due to progression disease (PD, 5 of which death), 1 due to bridge to ASCT, 2 due to Aes (namely sepsis with heart failure, acute renal failure and interstitial pneumonia, and acute hepatitis).

Best response rate was 43.8% (31.6% CR rate), with 10 CR, 4 PR, 5 stable diseases (SD) and 13 PD. ORR was 37.5% (31.6% CR rate), with 10 CR, 2 PR, 2 SD and 18 PD.

No hematological toxicities occurred, while 15 patients developed at least 1 extra-hematological toxicity (overall 39 Aes). Ten patients (31.3%) developed at least 1 irAE for a total of 17 irAEs (two grade ≥ 3 and three judged as serious AE [SAE]): 1 patient developed 4 irAEs, 1 patient had 3 irAEs, 2 patients had 2 irAEs and 6 patients 1 irAE, respectively. Two out of these ten patients had an endocrinopathy (not the same irAE developed during ICI treatment) and none of them had familiarity for (auto)immune diseases. Five out of these ten patients achieved at least a PR (3 CR and 2 PR).

Complete irAEs description with grade in severity is reported in Table 1.

**Table 1:** Immune-related adverse events occurred during treatment with immune-checkpoint inhibitors.

Pt ID	irAE	Drug(s)	Grade	Action (ICI)	Outcome
#1	Thyrotoxicosis	Pembrolizumab	1	None	Resolved
#1	Hypothyroidism	Pembrolizumab	2	None	Controlled with drugs
#2	Acute renal failure	Pembrolizumab	3	Permanent suspension	Resolved
#2	Interstitial pneumonia	Pembrolizumab	1	Permanent suspension	Resolved
#3	Immune fever	Tislelizumab	1	None	Resolved
#4	Pancreatitis	Nivolumab (+ BV)	3	Permanent suspension of BV	Resolved
#4	Diabetes mellitus	Nivolumab (+ BV)	2	None	Controlled with drugs
#4	Thyrotoxicosis	Nivolumab (+ BV)	1	None	Resolved
#4	Hypothyroidism	Nivolumab (+ BV)	2	None	Resolved
#5	Hypothyroidism	Nivolumab (+ BV)	2	None	Resolved
#6	Acute hepatitis	Nivolumab (+ BV)	2	Permanent suspension	Resolved
#7	Hypersensitivity pneumonia	Nivolumab (+ BV)	1	None	Resolved
#8	Muscle pain	Nivolumab (+ BV)	1	Temporary interruption	Resolved
#9	Thyrotoxicosis	Pembrolizumab	1	None	Resolved
#9	Hypothyroidism	Pembrolizumab	2	None	Resolved
#9	Diffuse pain in the major joints	Nivolumab (+ BV)	2	None	Resolved
#10	Hypothyroidism	Nivolumab (+ BV)	2	None	Resolved

*BV* brentuximab vedotin, *ICI* immune checkpoint inhibitor, *irAE* immune-related adverse event, *pt* patient.

All irAEs resolved beside a post-thyroiditis immune-based hypothyroidism and diabetes mellitus which was not autoimmune (both chronic and controlled with therapy). One SAE which was constituted by multi-organ failure led to patient death due to heart failure even if his irAEs (acute renal failure and interstitial pneumonia which caused hospitalization) were resolved.

Median time to presentation of irAEs was 69 days (range 0–407) with a median resolution time of 16 days (range 0–98). No late irAEs (i.e. after end of treatment) occurred. No statistically significant difference in irAEs frequency resulted between different ICIs (p = 0.181), histologies (p = 0.210) and outcomes (p = 0.158 for best response; p = 0.722 for ORR; p = 0.377 for deaths). No correlations were found between patients’ characteristics (age, gender, autoimmune diseases) and irAE occurrence.

Ten out of 17 (58.8%) irAEs were referred to endocrine glands. In particular, five patients developed sudden-onset hypothyroidism at different times (from 16 to 44 weeks after ICI starting). One PMBCL on pembrolizumab had an initial thyrotoxicosis at 6 weeks turning to overt hypothyroidism after 9 weeks. All the 5 subjects were asymptomatic. Two of them had positive anti-thyroid autoantibodies (anti-thyroid peroxidase and anti-thyroglobulin), whereas none had detectable anti-TSH (thyroid-stimulating hormone) receptors. All hypothyroid subjects started l-tyroxine therapy lifelong. At the last follow up, 3 out of the 5 patients with endocrine-irAE were in continuous CR, 1 still in PR and 1 had a relapse after an initial PR.

**Figure 1:**
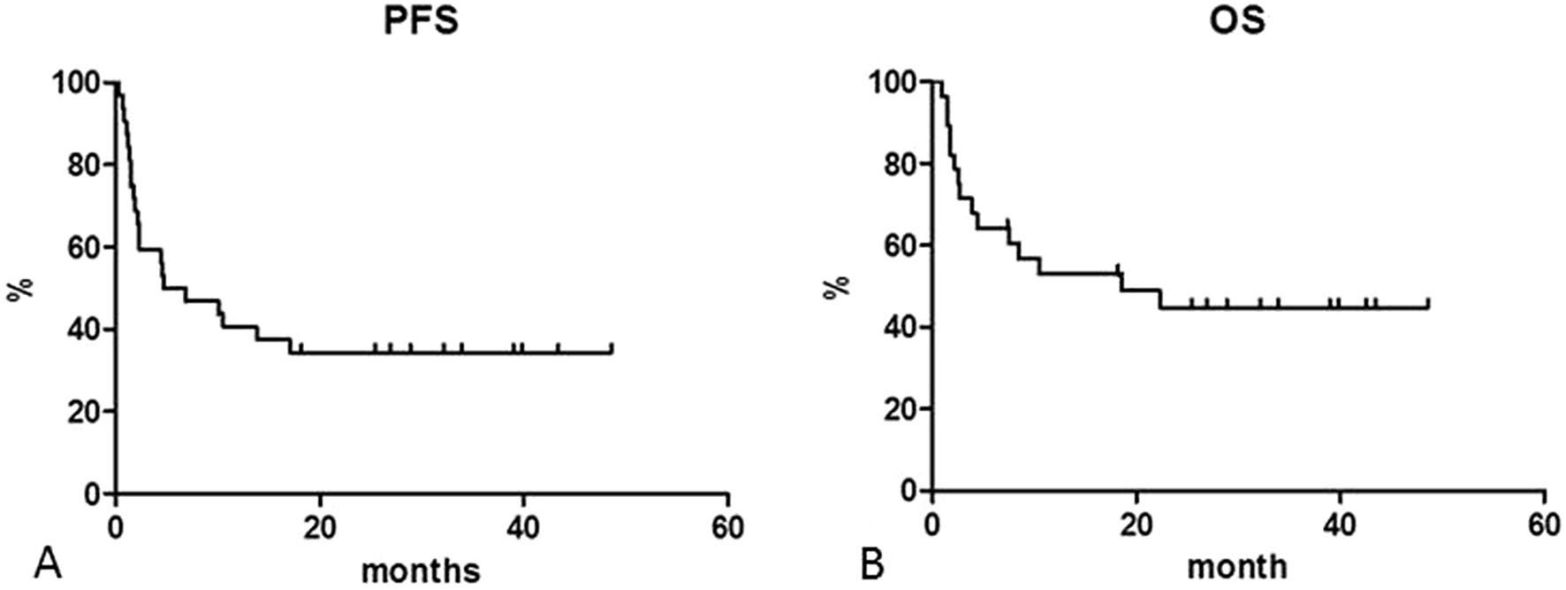
Progression free survival (PFS) (**A**) and overall survival (OS) (**B**).

**Figure 2:**
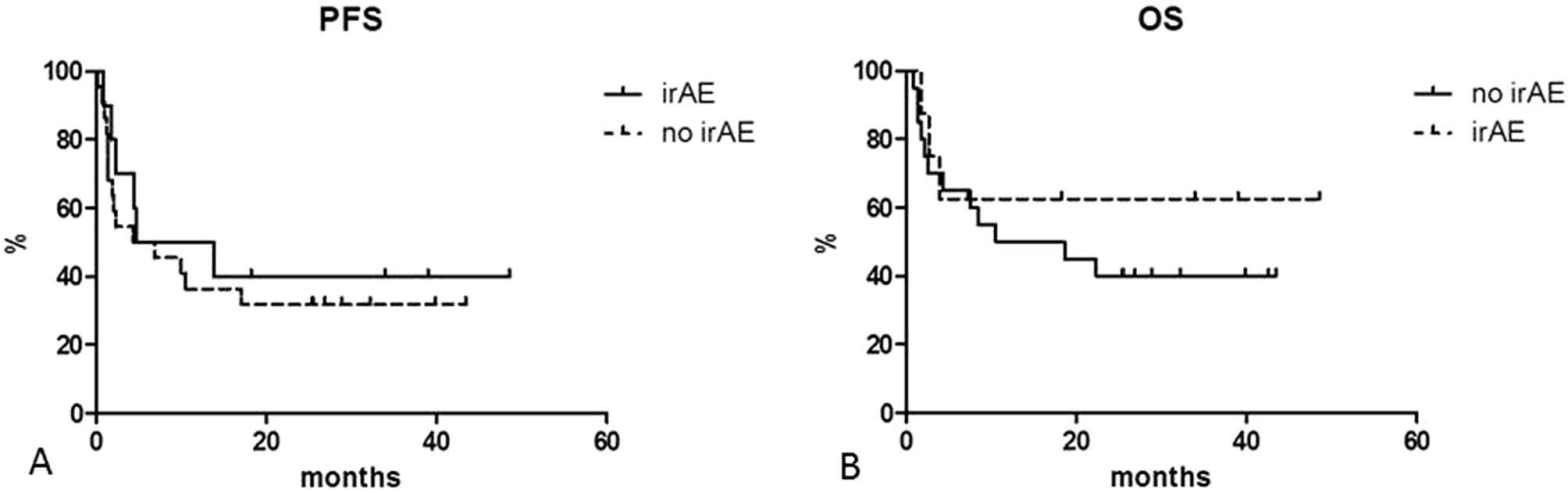
Progression free survival (PFS) for patients who developed an immune related adverse event (irAE) and for who did not (p = 0.5442) (**A**) and overall free survival (OS) for patients who developed an immune related adverse event (irAE) and for who did not (p = 0.4718) (**B**).

At a median follow up of 48.0 months, DFS was 100% at 24.0 months. PFS was 40.6% at 12 months, 34.4% at both 24.0 and 48.6 months (median reached at 5.8 months) (Fig. 1A). OS was 52.9% at 12 months. With 15 deaths, OS was 44.8% at both 24.0 and 48.6 months (median reached at 18.6 months) (Fig. 1B). We estimated PFS for patients who developed an irAE (40.0% at 24 months) and for who did not (31.8% at 24 months): the curves did not differ (p = 0.5442) (Fig. 2A). OS for the two groups did not differ (at 24 months 40.0% and 62.5% for patients without and with irAE, respectively; p = 0.4718), but median for who developed an irAE was not reached (Fig. 2B).

## Discussion

Efficacy and safety findings coming from clinical trials indicate that ICIs have potential to provide substantial clinical benefit in heavily pretreated patients with NHL, particularly given the lack of effective alternatives. Currently, only pembrolizumab is approved for R/R PMBCL by FDA but other ICIs are under investigation worldwide as single agents or in combination.

Recognizing and characterizing treatment-related Aes represent a cornerstone of determining the value of NHL treatments. A shortage of high-quality and reliable AE data, even from clinical trials, has prompted a call for more rigorous standards of AE reporting^13–16^. The advent of ICIs likely adds considerable challenge to this effort as the toxicities related to ICIs are peculiar and different from what previously observed with the other antineoplastic agents. In fact, irAEs may involve almost every organ and are unpredictable, sometimes permanent, and occasionally fatal^17,18^.

The increase in immune response caused by ICI can results in both disease regression and irAEs and, in fact, some studies reported a connection between their occurrences. In details, irAEs occurrence was related to both a longer OS and tumor regression^19–21^. In oncologic patients, namely affected by small cell lung carcinoma, an early occurrence of irAEs nivolumab-related was associated with better PFS and ORR^22^. This suggested that endocrine irAEs may be a symptom of an augmented immune response against neoplastic cells. In our study population, ICIs efficacy was not influenced by irAEs occurrence and patients’ outcomes were preserved: improving or worsening of response from best response to ORR; best and overall response rates did not significantly differ between patients who developed an irAEs and those who did not. PFS and OS at 24 months were higher in patients who developed an irAE (40% and 62.5%, respectively) than in those who did not (31.8%). These differences were not significant, but data supported the hypothesis that the highest activity of ICIs is associated with irAEs occurrence, which may be considered a clinical biomarker for ICI response^10^. To note, median OS for who developed an irAE was not reached.

A recent meta-analysis demonstrated that the frequency of each type of irAE depends on tumor type^23^, thus specific data on NHL are needed. In our study there was no difference among different NHL but probably a larger sample size is needed to confirm this association also in lymphomas.

Now that single-agent and combination ICIs regimens are coming for NHL, the timeliness and feasibility of irAEs recognition and diagnosis is crucial for the management of lymphoma patients in terms of both safety and efficacy. Single-center experiences in a real-world context with immunotherapy showed a significant higher irAE rates than those reported in prospective clinical studies^24–27^. To note that challenges in detection of irAEs seem to be caused by their heterogeneous manifestations, unpredictable timing, and clinical overlap with other conditions contribute^16,28^.

While most toxic effects of conventional chemotherapy and molecularly targeted therapies are readily diagnosed through medical history, physical examination, and laboratory data, irAEs appear far more heterogeneous. Our data showed that medical history cannot predict irAE onset and we did not find any relationship with histology, type of ICI or patients characteristics (with the limitation of a small sample size). The lack of specific immune biomarkers contributes to the challenges of capturing irAEs by clinicians. The American Society of Clinical Oncology and the National Comprehensive Cancer Network published clinical guidelines for irAEs diagnosis and management, but lymphomas-adapted guidelines are needed^7,29^.

Furthermore, to our knowledge, the accuracy of irAE diagnosis has not been evaluated and the observations in this regard in the literature are limited to a single report^30^.

Whether or not it is safe or necessary to resume checkpoint inhibition after a clinically significant irAE remains unclear. In our patients, no dose reduction was needed although on the other hand irAEs lead to ICI temporary suspension in one patient and withdrawn for two other subjects. To note, after AE resolution, one patient with muscle pain (grade 1) was retreated without new irAE occurrence; for other patients, in whom irAE caused suspension (acute hepatitis grade 2, acute renal failure grade 3 and interstitial pneumonia grade 1), the withdrawn was permanent. The clinician decision depended on the type of toxicity i.e. the organ concerned, the irAE grade and the possibility of using drugs that control the event (e.g. l thyroxine for hypothyroidism or beta-blockers for thyrotoxicosis).

Limitations of this analysis include the single-center setting. The strength of the study is its prospective design. In addition, this is the first report on irAE occurrence and management in NHL treated with ICIs as single agent or in combination.

The occurrence of irAEs in NHL seems lower than in solid neoplasia, especially for grade ≥ 3 ones^31^. On the contrary, endocrine irAEs are confirmed as the most frequent (58.8% of all the irAEs), as previously reported also for solid tumors^32,33^; thyroid dysfunction is the most common, which often presented as thyrotoxicosis. In our knowledge, therapy-related hypothyroidism is the unique irAEs characterized by well-defined laboratory values. In fact, the assessment of other relevant irAEs is complicated by the non-correlation with laboratory findings or by the fact that they may have non-immune causes.

Prompt consultation to the experts is of great importance and the grade of irAEs and patients’ disease conditions need to be carefully evaluated to decide the optimal measures. As irAEs could affect various organs, a multidisciplinary approach is critical, and it is important to organize a cooperative system within a hospital.

To our knowledge, our data are the first one collected prospectively on irAEs occurring in NHL treated with ICIs. Further studies are required to master this particular issue with the aim to provide clinical practice guidelines. With the increasing use of immunotherapy in lymphomas therapeutic algorithms, physicians must be aware about the drug-related irAEs, their recommended management, monitoring and about the best actions to be taken to avoid treatment discontinuation and, consequently, loss of patients’ response.

## Data Availability

The datasets used and analyzed during the current study are available from the corresponding authors on reasonable request.

## Abbreviations

AE: Adverse event
ASCT: Autologous stem cell transplantation
BV: Brentuximab vedotin
CR: Complete response
DFS: Disease free survival
ICI: Immune checkpoint inhibitor
irAE: Immune-related adverse event
NHL: Non-Hodgkin lymphoma
ORR: Overall response rate
OS: Overall survival
PD: Progressive disease
PD-1: Anti-programmed cell death 1
PD-L1: Anti-programmed cell death ligand 1
PR: Partial response
PFS: Progression free survival
PMBCL: Primary mediastinal B-cell lymphoma
R/R: Relapsed/refractory
SAE: Serious adverse event
